# Comparison of digital and analog [^68^Ga]Ga-PSMA-11 PET/CT for detecting post-prostatectomy biochemical recurrence in prostate cancer patients: a prospective study

**DOI:** 10.1038/s41598-024-65399-1

**Published:** 2024-07-01

**Authors:** Yong-il Kim, Dong Yun Lee, Changhwan Sung, Sang Ju Lee, Seung Jun Oh, Jungsu S. Oh, Shinkyo Yoon, Jae Lyun Lee, Bumjin Lim, Jungyo Suh, Juhyun Park, Dalsan You, In Gab Jeong, Jun Hyuk Hong, Hanjong Ahn, Choung-Soo Kim, Jin-Sook Ryu

**Affiliations:** 1 Department of Nuclear Medicine, Asan Medical Center, University of Ulsan College of Medicine, Seoul, Republic of Korea; 2 Department of Oncology, Asan Medical Center, University of Ulsan College of Medicine, Seoul, Republic of Korea; 3 Department of Urology, Asan Medical Center, University of Ulsan College of Medicine, Seoul, Republic of Korea

**Keywords:** Prostate cancer, Biochemical recurrence, Prostate-specific membrane antigen, Positron emission tomography, Detection rate, Urological cancer, Diagnostic markers

## Abstract

Digital positron emission tomography/computed tomography (PET/CT) has shown enhanced sensitivity and spatial resolution compared with analog PET/CT. The present study compared the diagnostic performance of digital and analog PET/CT with [^68^Ga]Ga-PSMA-11 in prostate cancer patients who experienced biochemical recurrence (BCR) after prostatectomy. Forty prostate cancer patients who experienced BCR, defined as serum prostate-specific antigen (PSA) concentrations exceeding 0.2 ng/mL after prostatectomy, were prospectively recruited. These patients were stratified into three groups based on their serum PSA levels. [^68^Ga]Ga-PSMA-11 was injected into each patient, and images were acquired using both analog and digital PET/CT scanners. Analog and digital PET/CT showed comparable lesion detection rate (71.8% vs. 74.4%), sensitivity (85.0% vs. 90.0%), and positive predictive value (PPV, 100.0% vs. 100.0%). However, digital PET/CT detected more lesions (139 vs. 111) and had higher maximum standardized uptake values (SUVmax, 14.3 vs. 10.3) and higher kappa index (0.657 vs. 0.502) than analog PET/CT, regardless of serum PSA levels. On both analog and digital PET/CT, lesion detection rates and interrater agreement increased with increasing serum PSA levels. Compared with analog PET/CT, digital PET/CT detected more lesions with a higher SUVmax and better interrater agreement in prostate cancer patients who experienced BCR after prostatectomy.

## Introduction

Prostate cancer is a common malignancy with increasing incidence in Western countries^1^. Biochemical recurrence (BCR) is defined as an increase in serum prostate-specific antigen (PSA) concentration after prostatectomy or radiation therapy without grossly visible recurrent lesions with conventional imaging^2^, indicating possible tumor recurrence^3^. Identifying the site of recurrence is crucial for appropriate treatment, especially metastasis directed therapy, and assessment of prognosis^4^. More sensitive imaging methods capable of detecting small lesions in patients with low serum PSA levels are therefore necessary for early diagnosis.

Prostate-specific membrane antigens (PSMAs) are type II membrane glycoproteins with extracellular, transmembrane, and intracellular components^5^. PSMAs are overexpressed in prostate cancer and can be targeted by radiotracer for positron emission tomography/computed tomography (PET/CT) imaging^6^. [^68^Ga]Ga-PSMA-11 is currently the most widely used PSMA-targeting radiotracer, especially in patients who experience BCR^7^. Analog [^68^Ga]Ga-PSMA-11 PET/CT showed lesion detection sensitivity below 50% in BCR patients with serum PSA concentrations ≤ 0.5 ng/mL^8^. By contrast, digital PET/CT, which employs silicon photomultiplier tubes (SiPM)^9^, is superior to analog PET/CT in terms of spatial resolution, sensitivity, and accuracy^10^. Studies using 2-[^18^F]fluoro-2-deoxy-D-glucose (FDG) or [^18^F]fluorocholine have confirmed that digital PET/CT has better lesion detectability than analog PET/CT^11,12^. To date, several retrospective studies comparing digital and analog PET/CT in the BCR diagnosis have been published^13,14^. However, no studies have directly compared digital and analog PET/CT in the diagnosis of BCR in a prospective study, a condition in which detectability is crucial.

This prospective study compared the diagnostic performances of digital and analog [^68^Ga]Ga-PSMA-11 PET/CT in patients who experienced BCR after prostatectomy. Factors compared included lesion detection rates, numbers of detected lesions, sensitivity, positive predictive value (PPV), uptake intensity, and interrater agreement.

## Results

### Patient characteristics

**Figure 1:**
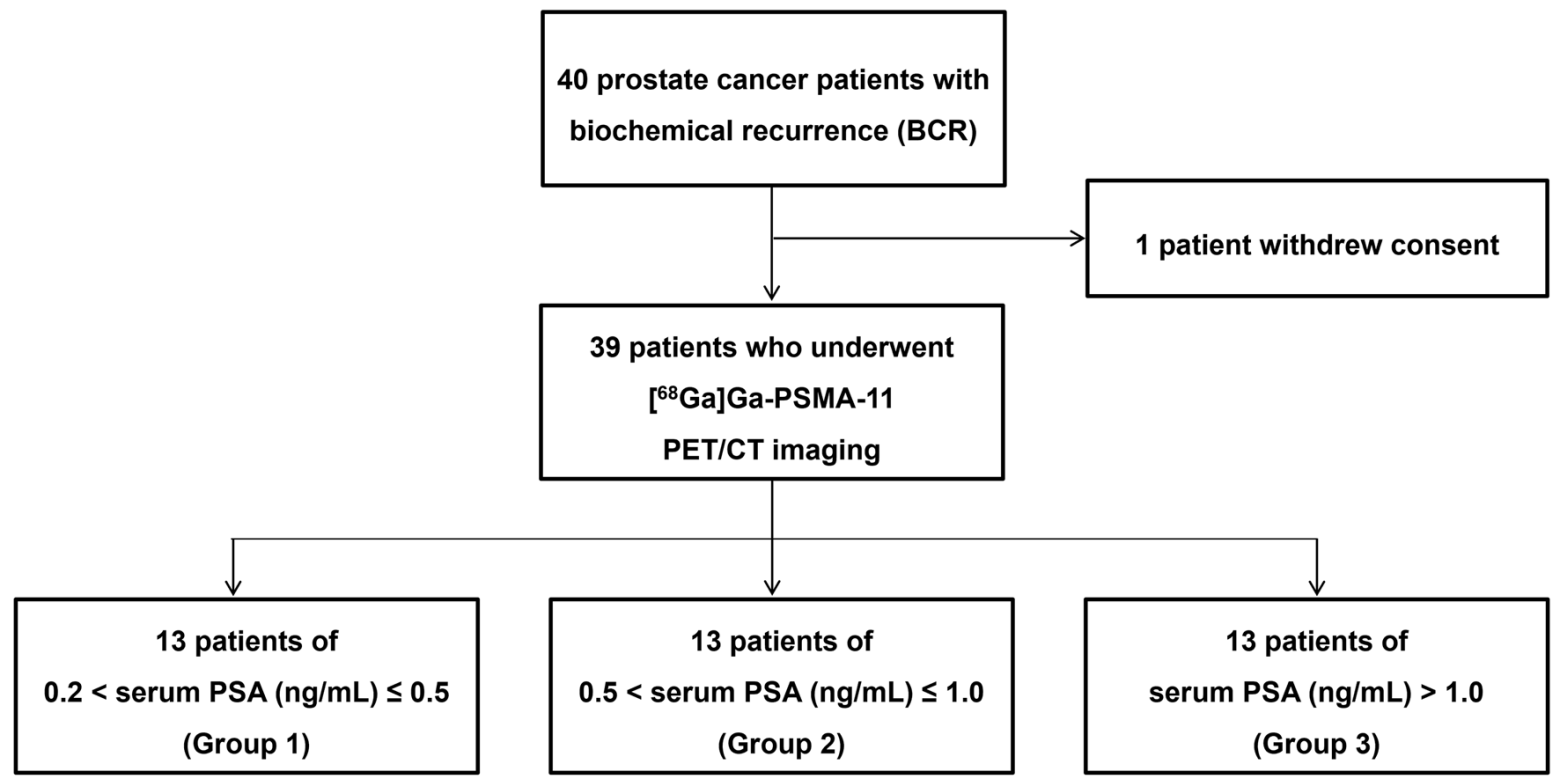
Flow chart of patients included in this study.

This study, conducted from April to August 2021, initially recruited 40 participants. One patient, however, withdrew consent, with results analyzed in 39 eligible patients (Fig. 1), of mean age 69.8 ± 8.0 years (range 55–90 years). Primary prostate cancer Gleason scores ranged from 7 to 9; however, Gleason scores for eight patients (20.5%) were unavailable as they did not undergo surgery at our institution. The mean serum PSA concentration at the time of work-up was 2.01 ± 2.71 ng/mL (range 0.23–10.62 ng/mL). Following prostatectomy, 17 patients (43.6%) received radiation therapy, 15 (38.5%) received hormone therapy, and three (7.7%) received chemotherapy. The patient profiles are summarized in Table 1.

**Table 1:** Demographic and clinical characteristics of patients with prostate cancer who experienced BCR after prostatectomy.

Characteristics	Values
Number	39
Age (year), mean ± SD (range)	69.8 ± 8.0 (55.0–90.0)
Gleason score, n (%)	
7	19 (48.7%)
8	2 (5.1%)
9	10 (25.6%)
Unknown	8 (20.5%)
Serum PSA level at work-up (ng/mL), mean ± SD (range)	2.01 ± 2.71 (0.23–10.62)
Group 1 (0.2 < serum PSA ≤ 0.5)	0.39 ± 0.10 (0.23–0.50)
Group 2 (0.5 < serum PSA ≤ 1.0)	0.72 ± 0.14 (0.52–0.93)
Group 3 (serum PSA > 1.0)	4.94 ± 3.05 (1.12–10.62)
Previous radiation therapy, n (%)	17 (43.6%)
Previous hormone therapy, n (%)	15 (38.5%)
Previous chemotherapy, n (%)	3 (7.7%)
Follow-up (months), mean ± SD (range)	15.0 ± 5.3 (3.7–25.0)

*PSA* prostate-specific antigen, *SUV* standardized uptake value.

### Lesion detection rate of [^68^Ga]Ga-PSMA-11 PET/CT

**Figure 2:**
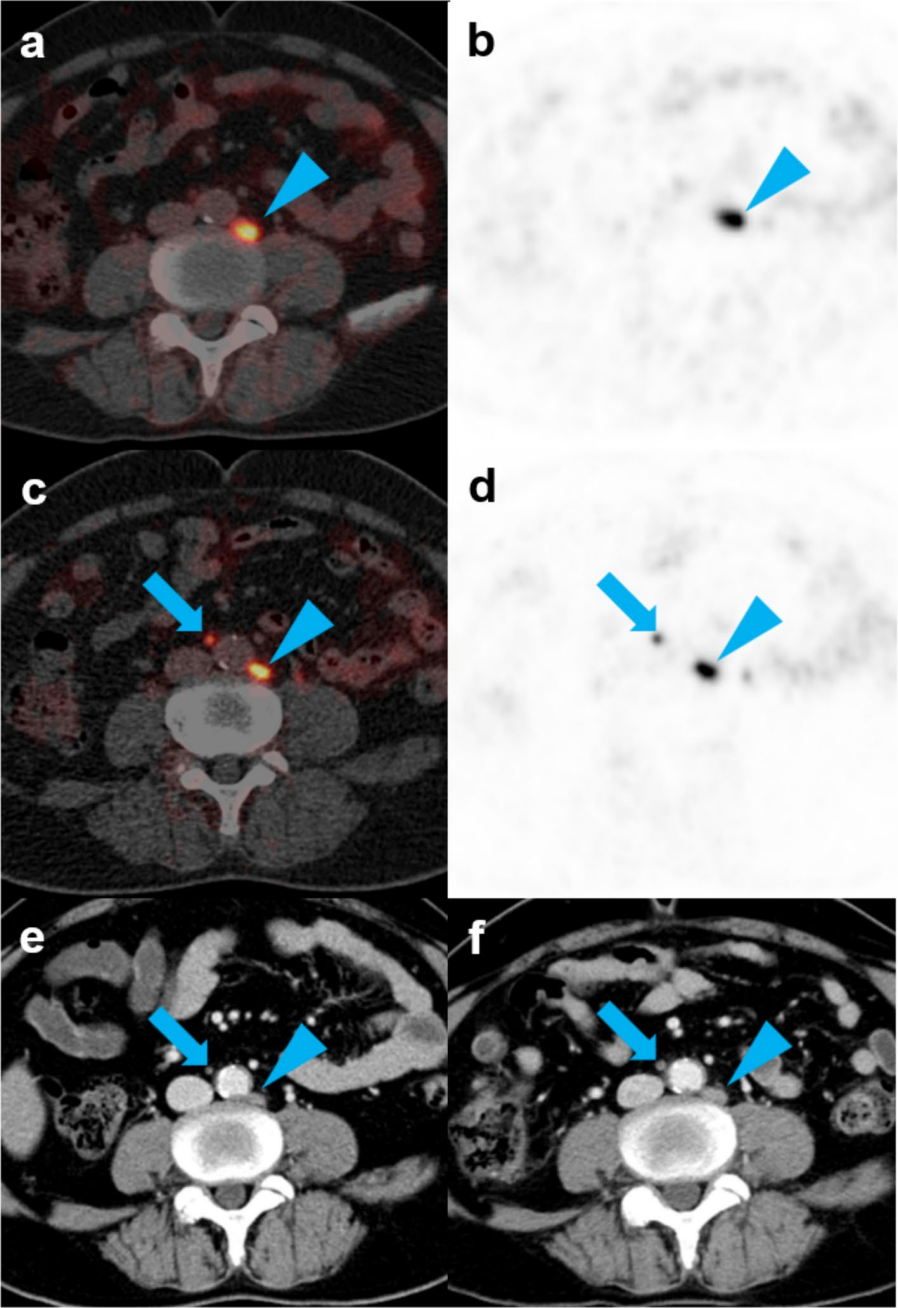
Detection of an additional metastatic lymph node by digital [^68^Ga]Ga-PSMA-11 PET/CT in a patient with prostate cancer who experienced BCR after prostatectomy. A 65-year-old man with prostate cancer was assessed by analog and digital [^68^Ga]Ga-PSMA-11 PET/CT after prostatectomy due to increased serum PSA level (5.02 ng/mL). (**a**,**b**) Analog PET/CT images showing a metastatic lymph node in the abdominal paraaortic area (arrowheads). (**c**,**d**) Digital PET/CT images showing a second small metastatic lymph node in the aortocaval area (arrows). (**e**) Subsequent contrast-enhanced CT, showing small lymph nodes in both the abdominal paraaortic and aortocaval areas. (**f**) Contrast-enhanced CT 6 months later showing an increase in size of both lymph nodes (arrows and arrowheads).

Analog PET/CT had an overall lesion detection rate of 71.8% (identifying 111 lesions in 28 patients), whereas digital PET/CT had an overall lesion detection rate of 74.4% (identifying 139 lesions in 29 patients). Digital PET/CT therefore detected 28 lesions not detected by analog PET/CT. Furthermore, digital PET/CT showed higher lesion detection rates in all three patient groups, stratified by serum PSA levels (Fig. 2). Furthermore, an increased lesion detection rate correlated with increased serum PSA concentrations (Table 2).

**Table 2:** Lesion detection rate and numbers of lesion identified by analog and digital [^68^Ga]Ga-PSMA-11 PET/CT.

Group	Analog PET/CT	Digital PET/CT
Total (39 patients)	71.8% (28 patients, 111 lesions)	74.4% (29 patients, 139 lesions)
Group 1 (13 patients)	38.5% (5 patients, 6 lesions)	38.5% (5 patients, 7 lesions)
Group 2 (13 patients)	76.9% (10 patients, 13 lesions)	84.6% (11 patients, 20 lesions)
Group 3 (13 patients)	100.0% (13 patients, 92 lesions)	100.0% (13 patients, 112 lesions)

*PSMA* prostate-specific membrane antigen, *PET/CT* positron emission tomography/computed tomography.

### Sensitivity and PPV of [^68^Ga]Ga-PSMA-11 PET/CT

Final diagnoses for standard of truth were available for 24 (61.5%) of the 39 patients, including 20 true positives and four true negatives, whereas 15 (38.5%) were inconclusive for standard of truth. Confirmation of the true positive lesions was based on a reduction in PSA concentration following radiation treatment of the prostatectomy bed (10 patients), lesion detection on follow-up bone scans (four patients) or follow-up CT/MRI (four patients), and surgery (two patients). Analog PET/CT had a sensitivity of 85.0% (17/20) and a PPV of 100.0% (17/17), whereas digital PET/CT had a sensitivity of were 90.0% (18/20) and a PPV of 100.0% (18/18) (Table 3).

**Table 3:** Sensitivity and PPV of analog and digital [^68^Ga]Ga-PSMA-11 PET/CT in patient-based analyses.

Parameters	Analog PET/CT	Digital PET/CT
Sensitivity	85.0% (17/20 patients)	90.0% (18/20 patients)
Group 1	80.0% (4/5)	80.0% (4/5)
Group 2	66.7% (5/7)	83.3% (6/7)
Group 3	100.0% (8/8)	100.0% (8/8)
PPV	100.0% (17/17 patients)	100.0% (18/18 patients)
Group 1	100.0% (4/4)	100.0% (4/4)
Group 2	100.0% (5/5)	100.0% (6/6)
Group 3	100.0% (8/8)	100.0% (8/8)

*PPV* positive predictive value.

### Comparison of quantitative parameters determined by analog and digital [^68^Ga]Ga-PSMA-11 PET/CT

**Figure 3:**
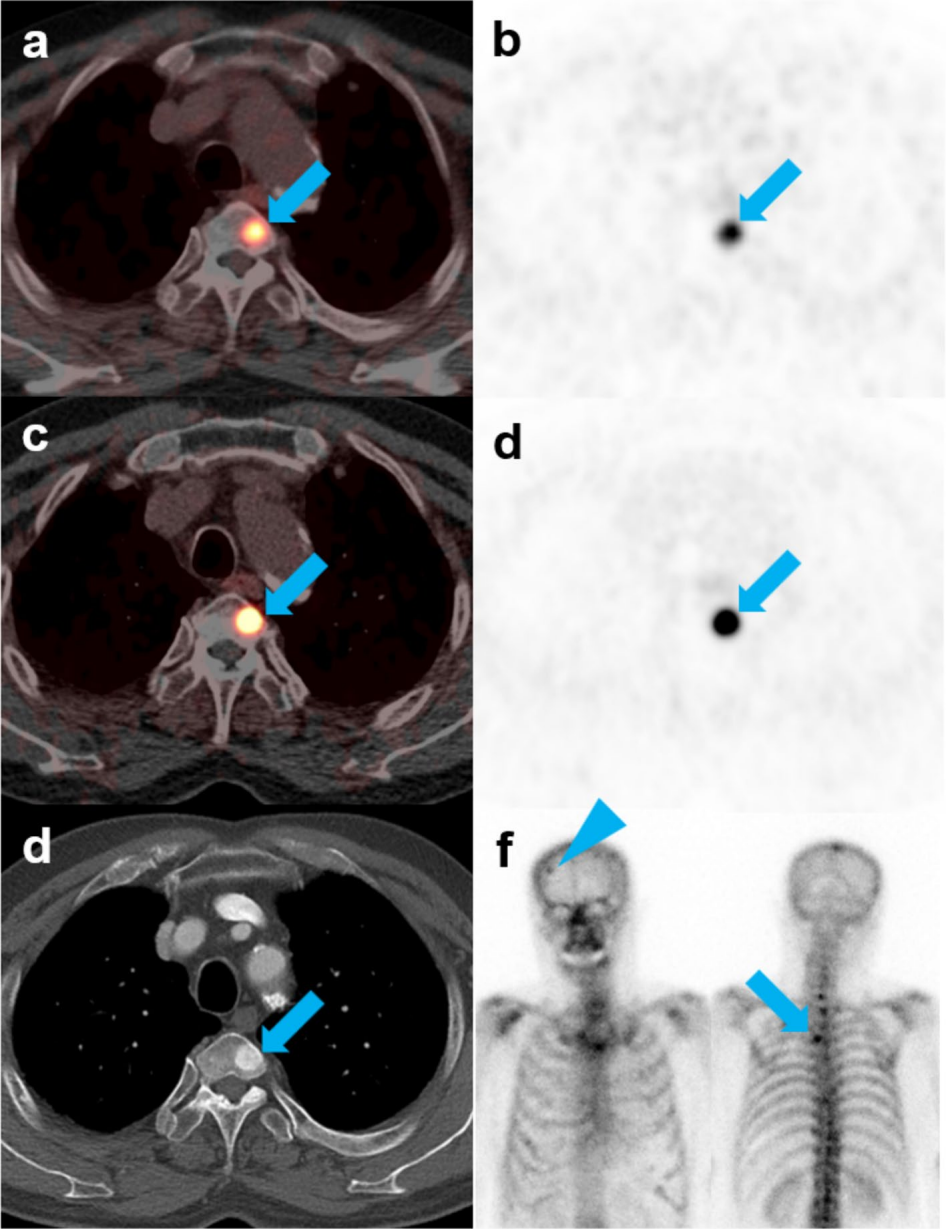
Detection of bone metastases by analog and digital [^68^Ga]Ga-PSMA-11 PET/CT in a patient with prostate cancer who experienced BCR after prostatectomy. A 73-year-old man with prostate cancer was assessed by analog and digital [^68^Ga]Ga-PSMA-11 PET/CT after prostatectomy due to increased serum PSA level (1.12 ng/mL). (**a**,**b**) Analog PET/CT images showing a T4 body bone metastasis with SUVmax of 32.5 (arrows). (**c**,**d**) Digital PET/CT images showing a T4 body bone metastasis with an SUVmax of 45.2 (arrows). (**e**) Contrast-enhanced CT showing a T4 body bone metastasis with osteosclerotic changes (arrow). (**f**) Follow-up bone scan showing bone metastases with increased uptake in the T4 (arrow) and right frontal bone of the skull (arrowhead).

Ninety-seven lesions identified on both analog and digital PET/CT scans were compared. Median SUVmax was significantly higher on digital than on analog PET/CT across all lesions (14.3 vs. 10.3, *p* < 0.001) and in lesions of patients with PSA concentrations > 0.5 ng/mL but ≤ 1 ng/mL (7.3 vs. 6.5, *p* = 0.003) and PSA > 1.0 ng/mL (16.1 vs. 11.1, *p* < 0.001). Median SUVmax was also significantly higher on digital than on analog PET/CT in the prostatectomy bed (14.5 vs. 9.1, *p* = 0.008), lymph nodes (13.3 vs. 8.8, *p* < 0.001), and bones (16.9 vs. 13.3, *p* < 0.001) (Fig. 3). By contrast, the SUVmean of the liver was significantly lower on digital than on analog PET/CT (3.7 vs. 4.1, *p* < 0.001) (Table 4).

**Table 4:** Quantitative measurements of analog and digital [^68^Ga]Ga-PSMA-11 PET/CT.

Location	Number	Analog PET/CT SUVmax	Digital PET/CT SUVmax	*p*-value
Total	97	10.3 (5.0–17.2)	14.3 (6.6–23.9)	< 0.001*
Subgroup analysis 1				
Group 1	4	4.5 (3.9–7.7)	5.5 (3.8–11.3)	0.375
Group 2	11	6.5 (4.2–10.9)	7.3 (5.7–18.5)	0.003*
Group 3	82	11.1 (5.1–19.8)	16.1 (8.0–28.2)	< 0.001*
Subgroup analysis 2				
Prostatectomy bed	8	9.1 (5.6–12.2)	14.5 (6.9–16.8)	0.008*
Lymph node	55	8.8 (4.7–14.6)	13.3 (6.5–22.0)	< 0.001*
Bone	34	13.3 (5.3–27.4)	16.9 (7.8–31.3)	< 0.001*
Liver SUVmean (reference)	39	4.1 (2.7–8.0)	3.7 (1.9–6.8)	< 0.001*

*SUVmax* maximum standardized uptake value, *SUVmean* mean standardized uptake value.

*Statistically significant (*p* < 0.05).

### Interrater agreement for interpretation of [^68^Ga]Ga-PSMA-11 PET/CT results

For the analog PET/CT, moderate agreement was observed between the two interpreters (κ = 0.5026, standard error = 0.1031), whereas the digital PET/CT showed substantial agreement (κ = 0.6566, standard error = 0.0955). The levels of interrater agreement on both analog and digital PET/CT increased with increasing serum PSA concentrations (Table 5).

**Table 5:** Interrater agreement for analog and digital [^68^Ga]Ga-PSMA-11 PET/CT.

Characteristics	Lesion number	Weighed Kappa (95% CI)	Standard error
Analog PET/CT	102	0.5026 (0.3004 to 0.7047)	0.1031
Group 1	17	0.0838 (− 0.2347 to 0.4024)	0.1625
Group 2	13	0.3810 (− 0.1230 to 0.8849)	0.2571
Group 3	72	0.6364 (0.3073 to 0.9654)	0.1679
Digital PET/CT	109	0.6566 (0.4694 to 0.9547)	0.0955
Group 1	15	0.3363 (− 0.1352 to 0.8078)	0.2401
Group 2	22	0.6271 (0.3029 to 0.9513)	0.1654
Group 3	72	0.6522 (0.2024 to 1.0000)	0.2950

*CI* confidence interval.

## Discussion

The present study was designed to compare the diagnostic performance of digital and analog PET/CT imaging using [^68^Ga]Ga-PSMA-11 in detecting lesions in patients who experienced BCR after prostatectomy. Digital PET/CT was found to provide a higher lesion detection rate, a higher SUVmax, and better interrater agreement than analog PET/CT. To our knowledge, this study is the first to directly compare the results of digital and analog PET/CT using [^68^Ga]Ga-PSMA-11.

The superiority of digital to analog PET/CT was likely due to the advanced time-of-flight (TOF) technology in the former. Digital PET showed a higher count rate performance, providing an improved TOF time resolution. The digital (Siemens Biograph Vision 600) and analog (GE Discovery 690) PET devices in the present study displayed time resolutions of about 210 and 550 picoseconds, respectively. The enhanced time resolution of the digital device allowed for more effective noise reduction and faster scans, improving its sensitivity^15,16^. Additionally, the extended axial field of view of the digital PET scanner further contributed to this increased sensitivity. Digital PET uses a smaller LSO crystal (3.2 mm) than analog PET (> 4 mm) and employs SiPM instead of photomultiplier tube (PMT), leading to better spatial resolution^17,18^. In the present study, the spatial resolutions of digital and analog PET were approximately 1.8 mm and 2.8 mm, respectively, when point spread function based reconstruction methods were applied. Moreover, digital PET employed a smaller sized post-smoothing Gaussian filter (2 mm full width at half maximum [FWHM]) than analog PET (4 mm FWHM). The detection rate of the analog PET/CT device used in the present study was in good agreement with previous results^19,20^.

To adhere to existing guidelines, [^68^Ga]Ga-PSMA-11 PET/CT imaging was performed 50–100 min after intravenous injection of 1.8–2.2 MBq/kg [^68^Ga]Ga-PSMA-11^21,22^. The lesion detection rate of analog PET/CT relative to serum PSA concentrations in the present study was in good agreement with previous findings^20,23^. Compared with analog PET/CT, digital PET/CT identified 28 additional lesions, enabling the detection of an additional patient with positive lesions. Most of the additional lesions detected by digital PET/CT were small in size. Conventional [^68^Ga]Ga-PSMA-11 PET/CT has been reported to modify treatment strategies and determine prognoses in over 50% of patients^24,25^. Enhanced lesion detection by digital PET/CT may improve the management of patients with postoperative BCR, for example, by determining the need for salvage radiation therapy^26^. Consequently, increased ability of digital PET/CT to detect lesions could lead to improved patient outcomes compared with analog PET/CT.

Digital PET/CT yielded higher SUVmax values than analog PET/CT across all lesions, serum PSA groups, prostatectomy beds, lymph nodes, and bones. By contrast, digital PET/CT yielded significantly lower liver SUVmean than analog PET/CT, indicating that the tumor-to-background ratio is much better in digital than in analog PET/CT. Although a prior study using 2-[^18^F]FDG also found that SUVmax was higher in digital than in analog PET/CT^27^, another study with matched pairs of digital and analog [^68^Ga]Ga-PSMA-11 PET/CT scans revealed no significant differences^13^. Taken together, these findings suggest the need for further studies evaluating SUVmax values determined by digital PET/CT.

The improved uptake and superior spatial resolution of digital PET/CT can affect an interpreter’s confidence level and agreement among interpreters. The present study found that digital PET/CT had substantial interrater agreement, whereas analog PET/CT had moderate agreement. Notably, digital PET/CT showed better agreement than analog PET/CT in patients with serum PSA concentrations ≤ 1.0 ng/mL. Similarly, a prior study of digital 2-[^18^F]FDG PET/CT showed better lesion detection and sharpness than analog 2-[^18^F]FDG PET/CT^28^. By contrast, a study comparing digital and analog [^68^Ga]Ga-PSMA-11 PET/CT found considerable agreement for digital PET/CT but near-perfect agreement for analog PET/CT, indicating a need for further investigation^14^. Moreover, subgroup analysis based on serum PSA level in the present study showed that interrater agreement was lower in lesions with lower uptake.

The present study had some limitations. It included a small number of patients from a single-center. In addition, due to the small sample size and for patient’s convenience, analog and digital PET/CT images were sequentially acquired after a single [^68^Ga]Ga-PSMA-11 injection, with digital PET/CT always being second in line. Therefore, differences in uptake time may have influenced the comparison between the two methods and may partly account for the found differences between analog and digital PET/CT. This fixed scanning order is a significant limitation of our study, as it introduces a potential bias that favors digital PET/CT due to the longer incubation period before scanning. Furthermore, as the composite standard of truth based on every 6 months of follow-up is a weak comparator, the sensitivity and PPV of our results should be interpreted with caution. Lastly, the two types of PET/CT scanners were manufactured by different companies. Although we attempted to match the imaging modalities as closely as possible, some differences in CT and PET parameters and reconstruction methods exist. Different parameters and reconstruction methods can cause differences in SUV^29^. Future prospective studies involving larger patient populations in multiple centers are warranted.

Compared with analog PET/CT, digital PET/CT can demonstrate superior performance in detecting more lesions with a higher SUVmax and can exhibit better interrater agreement in prostate cancer patients who experienced BCR after prostatectomy. These findings suggest that [^68^Ga]Ga-PSMA-11 digital PET/CT may become a more efficient method for identifying BCR lesions.

## Methods

### Study design and participant selection

This prospective, non-randomized, single-center, open-label feasibility study enrolled 40 patients exhibiting BCR following prostatectomy. BCR was defined as a postoperative PSA concentration > 0.2 ng/mL on at least two occasions, including one test performed within 1 month prior to providing consent to participate. All patients underwent standard imaging tests such as abdominopelvic CT and bone scans to confirm the absence of metastatic lesions. To evaluate the correlations between recurrent lesion detection rates and serum PSA concentrations^30^, the patients were stratified into three groups based on their serum PSA concentrations at screening: PSA > 0.2 ng/mL but ≤ 0.5 ng/mL; PSA > 0.5 ng/mL but ≤ 1 ng/mL; and PSA > 1.0 ng/mL.

Upon enrollment, patients were slated to undergo analog and digital PET/CT scanning after intravenous injection of [^68^Ga]Ga-PSMA-11. Two nuclear physicians with knowledge of the patient’s clinical history and previous image results reported the consensus imaging findings to the referring physician. Patients were clinically followed up for at least 6 months by additional imaging modalities, including CT, magnetic resonance imaging (MRI), and bone scan, in accordance with standard guidelines. Based on these clinical evaluations, patients underwent biopsy/surgery, localized radiation therapy, hormonal therapy, or combination therapy, as needed, with serum PSA measured at least every 6 months. At the final clinical follow-up, the PET/CT findings were evaluated using a composite standard of truth, including the results of histologic examinations, other imaging findings, and responses to treatment. Post hoc analyses conducted by blinded readers without access to clinical information were planned to compare the diagnostic performances of [^68^Ga]Ga-PSMA-11 PET/CT images obtained by analog and digital scanners.

This study was approved by the Institutional Review Board (IRB) at Asan Medical Center (no. 2021-0195), and all methods were performed in accordance with the relevant guidelines and regulations. Informed consent was obtained from all patients. The study protocol was registered at ClinicalTrials.gov (registration no. NCT04846894), and the first registration was done on 15/04/2021 (registration no. PSMA-S-04).

### Preparation of [^68^Ga]Ga-PSMA-11 and PET/CT imaging

The production of [^68^Ga]Ga-PSMA-11 adhered to the approved as hospital compounding drug, as sanctioned by the regional public health center at the institution's nuclear medicine department. All quality control procedures are performed according to European Pharmacopeia. [^68^Ga]Ga-PSMA-11 was prepared by eluting 3 mL of ^68^GaCl_3_ from a ^68^Ge/^68^Ga generator (iThemba Labs, South-Africa) using 0.6N HCl solution. Subsequently, 40 μg (42.2 nmol) of PSMA-11 and 1 mL of HEPES buffer complex solution (1 M HEPES buffer and NaOH mixture) were added to the GaCl_3_ solution, which was heated for 10 min at 100 °C. Following purification with a C18 SPE cartridge and pass-through a 0.22 μm sterile filter, [^68^Ga]Ga-PSMA-11 was collected in a sterile vial. Generally, radiochemical yield is > 70% and radiochemical purity is not less than 95%.

Patients were directed to ingest over 500 mL of water per hour prior to [^68^Ga]Ga-PSMA-11 PET/CT imaging. After an intravenous injection of 1.8–2.2 MBq/kg [^68^Ga]Ga-PSMA-11, patients were encouraged to drink an additional 500 mL of water within 30 min. PET/CT images were acquired 60 and 90 min after [^68^Ga]Ga-PSMA-11 injection using analog (GE Discovery 690; GE Medical Systems, Milwaukee, WI, USA) and digital (Siemens Biograph Vision 600; Siemens Healthcare, Erlangen, Germany) scanners, respectively. Imaging was performed from the vertex to the upper thigh in the caudocranial direction. CT images from each scanner were used to generate an attenuation map and localize the lesion. CT imaging parameters on analog PET/CT included 120 kVp, auto mA, 0.5 rotation time, and 3.75 mm slice thickness, whereas CT imaging parameters on digital PET/CT included 120 kVp, Care Dose 4D mA, 0.5 rotation time, and 3.00 mm slice thickness. Following CT imaging, PET imaging was performed in three-dimensional mode with a 500 mm field of view on both analog and digital scanners and 3 min of emission per bed position. Analog PET/CT images were reconstructed using the VPFX-S algorithm, consisting of four iterations, 18 subsets, a 4.0 mm field width at half maximum, and 192 × 192 matrices; whereas digital PET/CT images were reconstructed using the TrueX algorithm and time-of-flight methods, consisting of four iterations, five subsets, a 2.0 mm field width at half maximum, and 440 × 440 matrices.

### Image analysis

PET/CT images obtained were subject to both qualitative and quantitative analyses. Two nuclear medicine specialists (D. Y. L. & C. S.), blinded to the clinical findings and other imaging outcomes, independently assessed the [^68^Ga]Ga-PSMA-11 analog and digital PET/CT images using a predetermined report form. The certainty of detected lesions was assessed utilizing 5-grade scoring system (1—definitely negative, 2—probably negative, 3—possibly positive, 4—probably positive, and 5—definitely positive), and score of equal or more than 3 were considered positive. In case of discrepancies, a third nuclear medicine specialist (Y. K.) made the final decision. After that, we measured maximum standardized uptake values (SUVmax) of the positive recurrent lesions. SUVmax was calculated using the formula: SUV = (tissue radioactivity [Bq]/tissue weight [g])/(total radioactivity [Bq]/body weight [g]). Volumes-of-interest (VOIs) were drawn on the PET, CT, and fusion PET/CT images using the syngo.via software package (Siemens Healthcare, Erlangen, Germany), with maximum intensity projection (MIP) enabled and three-dimensional display settings (transaxial, coronal, and sagittal). As a reference, the mean SUV (SUVmean) of normal liver was determined in a VOI of about 10 cm^3^ in the right lobe of the liver.

### Outcome and statistical analysis

Categorical data were presented as counts with percentages, and continuous data as mean ± standard deviation (SD) or median and interquartile range (IQR). Initially, positive lesion detection rates (patient-based) and number of lesions (lesion-based) obtained by analog and digital [^68^Ga]Ga-PSMA-11 PET/CT imaging modalities were compared in all patients and in the three subgroups defined by serum PSA concentrations. Subsequently, a patient-based analysis using follow-up data was performed to determine the sensitivity and PPV of [^68^Ga]Ga-PSMA-11 PET/CT imaging findings. The SUVmax of analog and digital PET/CT scans of positive lesions were subsequently compared in relation to total lesions, serum PSA concentrations, and lesion locations using Wilcoxon's signed-rank test. Interrater agreement between the two interpreters based on serum PSA level was compared using Cohen's weighted kappa (κ) indices. A *p*-value less than 0.05 was considered statistically significant. All statistical analyses were performed using SPSS software (Version 18.0; SPSS Inc., Chicago, IL, USA) and MedCalc (Version 20.218; MedCalc Inc., Mariakerke, Belgium).

## Data Availability

The datasets generated during and/or analysed during the current study are available from the corresponding author on reasonable request.
